# Iron biofortification in maize by *ZmNAC78* is a promising and sustainable way to fight iron‐deficiency anaemia

**DOI:** 10.1002/ctm2.1538

**Published:** 2024-01-15

**Authors:** Qingguo Du, Wen‐Xue Li

**Affiliations:** ^1^ State Key Laboratory of Crop Gene Resources and Breeding, National Engineering Laboratory for Crop Molecular Breeding, Institute of Crop Sciences, Chinese Academy of Agricultural Sciences Beijing China

**Keywords:** biofortification, iron‐deficiency anaemia

Approximately one‐third of the global population is suffering from anaemia, with half of the cases being iron‐deficiency anaemia (IDA). IDA has become a global public health problem that particularly affects children, pregnant women and people in developing countries.^1^, ^2^ In China, the government is aiming at controlling anaemia prevalence in children under 5 years old and pregnant women below 10% by 2030.^3^ Although iron supplements can quickly improve iron nutrition in human, the costs are relatively high. Increasing the iron content in daily consumed crops would be a more fundamental and cost‐effective approach to improve the iron nutrition of a large population. Recently, we identified *ZmNAC78* as a key gene regulating iron loading into maize kernels and cultivated maize varieties with both high yield and high iron concentrations in kernels using a molecular marker developed from *ZmNAC78*.^4^ Our results provide novel and practicable gene resources to reduce IDA in developing countries.

## 1 *ZmNAC78* REGULATES IRON LOADING INTO MAIZE KERNELS

Maize ranks first in total production among major staple cereals in the world and is mainly used as a staple food in sub‐Saharan Africa, in where the risk of iron‐deficiency is much greater than in other regions.^5^ However, the iron content in maize grains is negatively correlated with yield.^4^ In addition, the processes of iron loading into crop kernels are almost completely unknown, which greatly limits the developing maize varieties with both iron‐enriched kernels and high yield. In our research, we used the genotype data from 273 maize inbred lines and transcriptome data from six extreme materials and identified a candidate gene, *ZmNAC78*, regulating the iron content in maize. Overexpression of *ZmNAC78* significantly increased the iron concentrations in maize kernels to 70.5 mg per kilogram, which is more than 2 times greater than the current maize varieties used for production in China.

**FIGURE 1:**
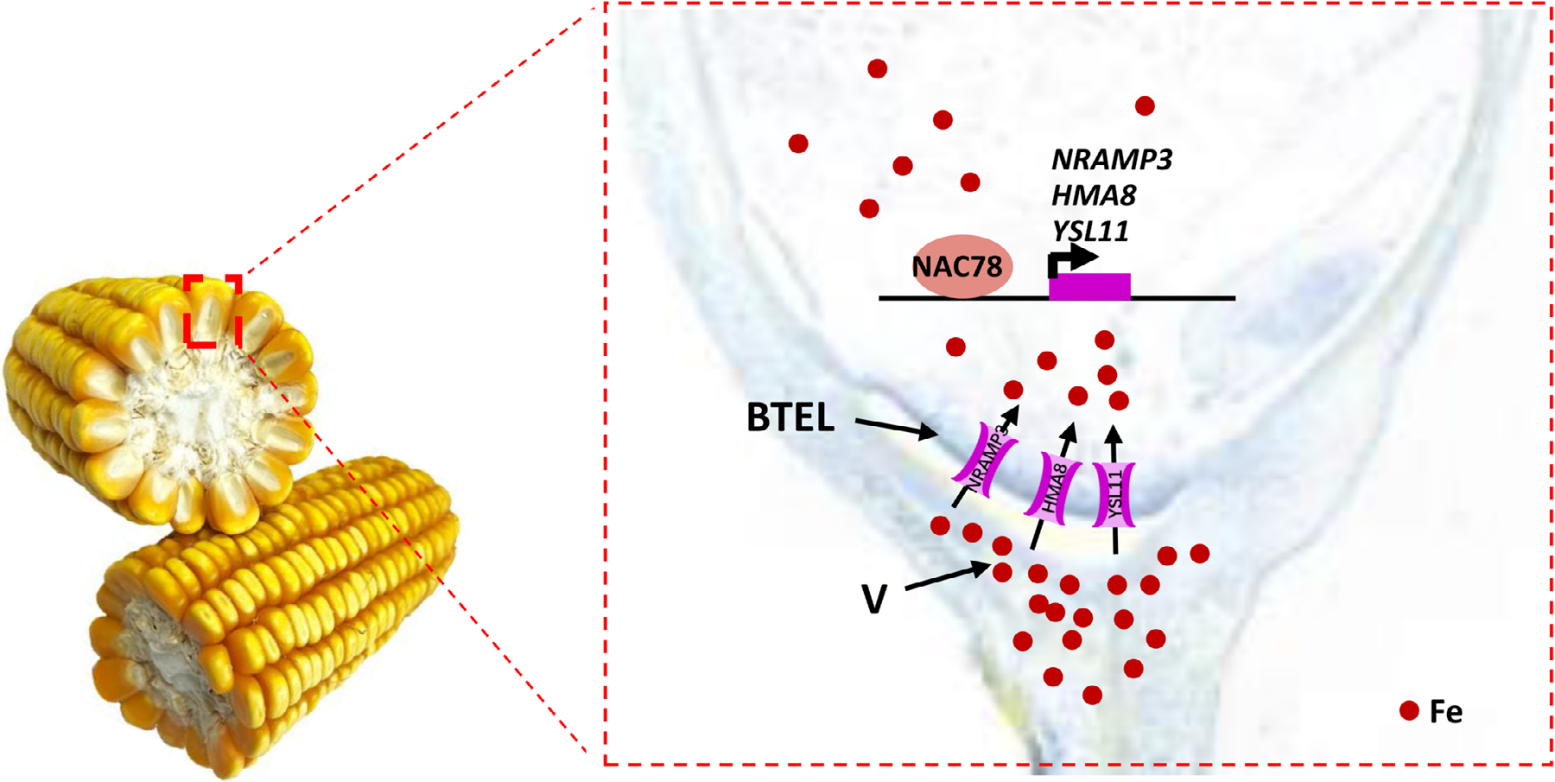
A proposed model illustrating the role of ZmNAC78 in regulating iron loading into maize kernels. BETL, basal endosperm transfer layer; V, vascular bundle.

We further analysed the molecular pathway for iron entry into maize grains. The results showed that *ZmNAC78* was preferentially expressed in the basal endosperm transfer layer (BETL), which is the only exchange surface between maternal and filial tissues in maize. ZmNAC78 could activate the expression of cation transporters *ZmHMA8*, *ZmYSL11* and *ZmNRAMP3*. In agreement with the expression patterns of *ZmNAC78*, these three genes were also preferentially expressed in the early stage of kernel development and in BETL. Stop‐gained *ZmNRAMP3* and *ZmHMA8* significantly reduced iron concentrations in maize kernels. These results suggest that ZmNAC78 and cation transport proteins together form a molecular switch controlling iron loading into maize grains (Figure 1). As pointed by the reviewers, the way in which iron gets into grain is almost completely unknown, and identifying, for what they believe is probably the very first time, a gene involved in this process is a ‘big deal’.

## 2 *ZmNAC78* CAN BE USED FOR BREEDING OF IRON‐ENRICHED MAIZE VARIETIES

To explore the application of *ZmNAC78* in breeding, the inbred lines were grouped into haplotype1 (Hap1) and haplotype2 (Hap2). The abundance of *ZmNAC78* and iron concentrations in kernels were significantly higher in Hap1 than in Hap2. According to the nucleotide polymorphisms of the *ZmNAC78* core promoter sequence, we developed a molecular marker to perform molecular marker‐associated selection of maize varieties with iron‐enriched kernels. To avoid the effects of environmental factors such as soil pH, five self‐breeding varieties, including three from Hap1 and two from Hap2 were planted alongside Zhengdan958 (the most widely grown commercial hybrid in China) in Yuanyang, Henan Province (pH 8.5) and in Nanning, Guangxi Zhuang Autonomous Region (pH 6.4). The average iron concentrations in Hap1 varieties were higher than in Hap2 varieties, with an average increase of 25.82% to 33.91%. Encouragingly, the self‐breeding Variety1 with Hap1 type showed both higher iron concentrations and grain yield compared with Zhengdan958. These results indicated that *ZmNAC78* is a practicable gene resource for iron biofortification in maize without reducing yield. The reviewer also noticed this and thinks that *ZmNAC78* is useful for the generation of iron‐enriched maize without pleiotropic effects.

## 3 FUTURE OUTLOOK

This research not only reveals the biological pathway of iron entry in maize grains but also provides new insights into how nutrients enter cereal crops with transfer cells such as wheat. To our knowledge, this research is the first report of the development of iron‐enriched maize varieties via molecular‐assisted breeding. We noticed that *ZmNAC78* overexpression had a meaningful contribution to iron concentrations in maize kernels grown in a latosol soil, and a relatively limited contribution in those grown in an alkaline cinnamon soil. Synergistic expression of *ZmNAC78* and genes related to iron uptake and translocation in maize should be a promising candidate to overcome the limitation. The plant‐based foods usually contain high phytic acid, which chelates cations, including iron, to form insoluble and nonabsorbable complexes in the upper gastrointestinal tract.^6^ There are two basic categories of industrial processing (dry and wet milling) employed for transforming maize into products for human consumption. The products and coproducts obtained from dry milling including nixtamalisation are used by the consumer. Nixtamalisation can activate phytase in the kernels to remove phytic acid. Exogenous application of phytase during industrial processing would make the plant‐based iron readily absorbable by humans. Thus, iron biofortification in crops be used as a promising and sustainable way to reduce IDA in developing countries.

## AUTHOR CONTRIBUTIONS

Qingguo Du: Writing‐original draft. Wen‐Xue Li: Writing‐review and editing.

## CONFLICT OF INTEREST STATEMENT

The authors declare no conflicts of interest.

## ETHICS STATEMENTS

Approval of the research protocol by an Institutional Reviewer Board: N/A.
